# The functions of nonsuicidal self-injury: converging evidence for a two-factor structure

**DOI:** 10.1186/s13034-015-0073-4

**Published:** 2015-09-28

**Authors:** E. David Klonsky, Catherine R. Glenn, Denise M. Styer, Thomas M. Olino, Jason J. Washburn

**Affiliations:** Department of Psychology, University of British Columbia, 2136 West Mall, Vancouver, BC V6T 1Z4 Canada; Harvard University, Cambridge, USA; Alexian Brothers Behavioral Health Hospital, Hoffman Estates, USA; Temple University, Philadelphia, USA; Alexian Brothers Behavioral Health Hospital, Northwestern University Feinberg School of Medicine, Chicago, USA

## Abstract

Research has identified more than a dozen functions of non-suicidal self-injury (NSI), but the conceptual and empirical overlap among these functions remains unclear. The present study examined the structure of NSI functions in two large samples of patients receiving acute-care treatment for NSI. Two different measures of NSI functions were utilized to maximize generalizability of findings: one sample (*n* = 946) was administered the Inventory of Statements About Self-injury (ISAS; Klonsky and Glenn in J Psychopathol Behav Assess 31:215–219, 2009), and a second sample (*n* = 211) was administered the Functional Assessment of Self-Mutilation (FASM; Lloyd et al. in Self-mutilation in a community sample of adolescents: descriptive characteristics and provisional prevalence rates. Poster session at the annual meeting of the Society for Behavioral Medicine, New Orleans, LA, 1997). Exploratory factor analyses revealed that both measures exhibited a robust two-factor structure: one factor represented Intrapersonal functions, such as affect regulation and anti-dissociation, and a second factor represented Social functions, such as interpersonal influence and peer bonding. In support of the two-factor structure’s construct validity, the factors exhibited a pattern of correlations with indicators of NSI severity that was consistent with past research and theory. Findings have important implications for theory, research, and treatment. In particular, the two-factor framework should guide clinical assessment, as well as future research on the implications of NSI functions for course, prognosis, treatment, and suicide risk.

## Introduction

Non-suicidal self-injury (NSI) refers to the intentional destruction of one’s own body tissue without suicidal intent and for purposes not socially sanctioned (ISSS [13]). Approximately 4–6 % of adults in the general population report having engaged in NSI at least once [16, 20], and this figure increases to approximately 14–18 % in community samples of adolescents and young adults [24, 25, 29, 32]. NSI is of concern due to its association with a variety of psychological disorders, as well as both its concurrent and prospective relationship to suicidal behavior [1, 2, 18, 20, 33].

Whereas early research tended to focus on psychosocial and diagnostic correlates of NSI, many studies from the last 10 years have addressed the functions of NSI [5, 14, 22, 27]. A functional perspective emphasizes variables that may be conceptualized as motivating or reinforcing the behavior [14]. Research on NSI functions has greatly advanced understanding of NSI. For example, it is now well established that affect regulation—using NSI to alleviate intense negative emotions—is the most common function of NSI, endorsed by more than 90 % of those who engage in the behavior [4, 15, 14]. It is also well documented that 50 % or more of those who self-injure endorse self-punishment, or self-directed anger, as a motivation for NSI [14], a pattern that has led subsequent studies to elucidate the role of self-criticism in NSI [12]. Many other NSI functions have also been identified including anti-dissociation (e.g., causing pain to stop feeling numb), anti-suicide (e.g., stopping suicidal thoughts), peer bonding (e.g., fitting in with others), interpersonal influence (e.g., letting others know the extent of emotional pain), and sensation seeking (e.g., doing something to generate excitement) [14, 17].

Despite the high endorsement of affective regulation functions of NSI, most individuals who self-injure endorse multiple functions [14, 17, 26]. Therefore, it is important to understand the extent to which different functions overlap or co-occur. For example, reducing negative feelings (affect regulation) may help reduce suicidal thoughts (anti-suicide), as well as reduce dissociation (anti-dissociation) for those who feel numb or unreal when overwhelmed by intense negative emotions. Similarly, using NSI to influence others (interpersonal influence) may include using the behavior to improve relationships with others who self-injure (peer bonding), as well as using NSI in social circles as an ‘extreme’ or exciting activity (sensation seeking). In addition, there is accumulating evidence that different NSI functions have different implications for treatment, prognosis, and suicide risk [17, 19, 27]. Thus, understanding the conceptual and empirical overlap among functions is critical both for theory development in research contexts and for case conceptualization and treatment planning in clinical contexts.

One study in particular has been influential in addressing covariation among NSI functions. Nock and Prinstein [26] administered the Functional Assessment of Self-Mutilation (FASM; [23]) to a sample of 89 adolescent patients with histories of NSI. The FASM is a self-report questionnaire that includes 22 reasons for engaging in NSI. Nock and Prinstein [26] utilized confirmatory factor analyses (CFA) to examine the structure of the 22 reasons and concluded that the motivations were best conceptualized as falling into one of four different categories: Automatic-Negative (use of NSI to reduce unpleasant internal states), Automatic-Positive (use of NSI to produce desirable internal states), Social-Negative (use of NSI to escape from interpersonal demands), and Social-Positive (use of NSI to gain attention or desirable responses from others). Importantly, Nock and Prinstein [26] also found a good fit for a two-factor model of NSI functions: Automatic and Social. This two-factor model fit the data as well as the less parsimonious four-factor model; however, the authors retained the latter on theoretical grounds.

The four-factor model advocated by Nock and Prinstein [26] has been extremely influential, as evidenced in part by a Google Scholar citation count exceeding 600. It is thus important to consider limitations of the evidence supporting the four-factor structure. First, the sample size was relatively small, reducing power to detect differences in fit between competing models (e.g., two-factor vs. four-factor). Second, some correlations between factors were high. For example, the Social-Negative and Social-Positive factors correlated .78, a magnitude high enough to suggest they represent the same latent factor [6]. Similarly, the Automatic-Negative and Automatic-Positive factors correlated .52, which is high considering that the low coefficient alphas for these two factors (.62 and .69, respectively) limit the extent to which these variables can correlate. Third, the Automatic-Negative factor consisted of just two items, which presents a challenge to its reliability and replicability. Perhaps as a consequence, in a subsequent study, one of the two Automatic-Negative items was switched to the Automatic-Positive factor for both empirical and conceptual reasons [28], leaving just a single item on the Automatic-Negative scale. Finally, Nock and Prinstein [26] utilized a CFA rather than an exploratory factor analysis (EFA). CFA is indeed useful for evaluating a theoretically derived structure. At the same time, because CFA requires identifying item-factor loadings a priori, the use of CFA places limits on the number and nature of factors that may emerge. Therefore, EFA, which places no such factor restrictions, may be especially appropriate for early stages of structural research (for elaboration see [8]).

Indeed, a recent spate of studies has examined the factor structure of the FASM and found solutions that diverge from that reported in Nock and Prinstein [26]. A study of a Chinese version of the FASM found that the four-factor structure reported by Nock and Prinstein [26] provided inadequate fit [21]. Two other studies of the FASM have found empirical support for a three-factor solution: (1) automatic, (2) social influence/communication, (3) peer identification/conformity. Specifically, Young et al. [34] found this structure utilizing principal components analysis of 170 15-year old students, and Dahlström et al. [7] found this structure using both EFA and CFA in 836 adolescents. Dahlstrom et al. also found excellent fit for a theoretically driven four factor solution consisting of one automatic factor and three social factors (social influence, peer identification, and avoiding demands).

The research described so far has focused on the structure of NSI functions as assessed by a particular measure, the FASM. Of course, any structure that emerges from research on this measure may reflect particular properties of the FASM rather than of NSI functions more generally. It is therefore important to note a separate line of research on NSI functions that has focused on another measure: the Inventory of Statements About Self-injury (ISAS; [17]). The ISAS is a self-report questionnaire consisting of 39 reasons for engaging in NSI, which are organized into 13 rationally derived functional scales. Klonsky and Glenn [17] utilized EFA to examine the structure of the 13 scales in a sample of 235 university students with histories of NSI and found that they were best conceptualized as representing two superordinate factors: Intrapersonal and Interpersonal functions. The Intrapersonal factor included self-focused functions, such as affect regulation and self-punishment, whereas the Interpersonal factor included other-focused functions, such as interpersonal influence and peer bonding. Klonsky and Glenn [17] concluded that these Intrapersonal and Interpersonal factors were conceptually equivalent to Nock and Prinstein’s [26] Automatic and Social factors, respectively. This two-factor structure was later further supported by a confirmatory factor analysis in a large (n = 529) Turkish sample of high school students with NSI histories [3].

However, two important limitations of both Klonsky and Glenn [17] and Bildik et al. [3] deserve note. First, both studies factor-analyzed the 13 ISAS scales rather than the 39 ISAS items. Thus, research has yet to empirically examine the structure of the ISAS at the item-level. Second, both studies utilized non-clinical samples; many participants may have engaged in infrequent or sub-clinical NSI, which may limit generalizability to treatment-seeking populations.

The present study was conceived to address ambiguity regarding the structure of NSI functions. Specifically, in two large samples of patients receiving acute-care treatment for NSI, we utilized EFA to investigate the structure of NSI functions as assessed by both the ISAS and the FASM. Use of two different measures helps ensure that findings will be generalizable, rather than artifacts of a particular questionnaire, and the large sample sizes provide sufficient power for item-level EFAs. In addition, this will be the first investigation of the structure of NSI functions to use large samples of patients. Based on findings from both Nock and Prinstein [26] and Klonsky and Glenn [17], we suspect a two-factor structure will best characterize NSI functions: Intrapersonal (Automatic) and Social (Interpersonal).^1^ However, because neither the FASM nor ISAS items have been examined using an exploratory approach in patient populations, and because recent studies on the FASM have produced both three and four-factor structures, we utilized EFA so as not to constrain the number and nature of functional factors that could emerge.

## Methods

### Participants

Participants included 1157 patients admitted to a NSI treatment program in a large behavioral health hospital over a 4 years period. The treatment program provides acute-care treatment for NSI, including inpatient, partial hospitalization, and intensive outpatient treatment. All participants reported a history of NSI, with more than half of participants (61.4 %) engaging in NSI in the week prior to admission. Common forms of NSI include cutting (92.5 %), scratching (63.3 %), head banging (37.2 %), preventing injuries from healing (37.2 %), tattooing for pain (33.5 %), burning skin (33.3 %), and pulling hair (23.8 %).

Participants received clinical diagnoses from an attending psychiatrist overseeing their treatment. Depressive disorders were the most common Axis I diagnosis (75.5 %), followed by anxiety (50.4 %), drug (29.4 %), eating (27.3 %), impulse control (26.8 %), bipolar (24.8 %), mood NOS (19.0 %), alcohol (16.7 %), posttraumatic stress (13.0 %), attention-deficit/hyperactivity (12.9 %), and psychotic (1.5 %) disorders. Nearly three-quarters (71.0 %) of participants were diagnosed with more than one Axis I disorder (Mean = 2.2 diagnoses, Standard Deviation [*SD*] = 1.0). Axis II disorders are not reported because they were not consistently evaluated by psychiatrists. Over one-third (37.4 %) of the sample indicated a history of suicidal behavior.

Participants were predominately female (89.4 %) and non-Hispanic white (72.1 %), with limited representation of Hispanic (6.2 %), African American (1.9 %), American Indian (<1 %), Asian (<1 %), and other ethnic groups; race/ethnicity was not reported for 18.7 % of the sample. Participant age ranged from 11 to 73 years with a mean age of 16.6 year (*SD* = 7.7); approximately two-thirds (65.9 %) of the sample were minors. Participants were hospitalized, on average, for less than 2 weeks (Mean = 12.5 days, *SD* = 13.4) on the inpatient unit, with slightly longer stays for partial hospitalization and intensive outpatient treatment (Mean = 16.1, *SD* = 11.0).

The ISAS was completed by 946 participants and a separate sample of 211 participants completed the FASM. No significant differences were found for demographic variables (age, gender, race/ethnicity) or for NSI behaviors between participants who completed the ISAS and the FASM (all *p*s > .05).

### Procedure

Patients were administered the ISAS or FASM along with other clinical measures during hospital admission for initial clinical assessment and to monitor clinical outcomes associated with treatment. The FASM was administered for the first year of data collection, at which point the FASM was replaced with the ISAS for the last 3 years to provide a more comprehensive assessment of NSI functions. These data were collected as part of routine clinical assessment for treatment purposes and no additional interaction with participants (including informed consent from participants or legal guardians) took place. The use of these pre-existing de-identified data for this research is exempt from the requirement for informed consent under 45 CFR 46.101(b)(4), and is also consistent with guidelines issued by the U.S. Department of Health and Human Services: http://answers.hhs.gov/ohrp/categories/1566). The process of de-identification followed the de-identification standard (45 CFR 164.514[a][b]) and was reviewed and approved by the Alexian Brothers Health System Institutional Review Board.

### Measures

#### ISAS

The ISAS [17] assesses 13 functions of NSI: affect regulation, anti-dissociation, anti-suicide, marking distress, self-punishment, autonomy, interpersonal boundaries, interpersonal influence, peer bonding, revenge, self-care, sensation seeking, toughness. Each subscale is assessed with three items rated on a scale from 0 = *not at all relevant* to 2 = *very relevant* to one’s experience of NSI. The ISAS has demonstrated structural and construct validity in both university and high-school students [3] [17] as well as good test–retest reliability in university students [9]. As discussed above, Klonsky and Glenn [17] grouped the ISAS subscales into two factors, which they termed: Intrapersonal and Interpersonal.

#### FASM

The FASM [23] includes 22 items assessing reasons for NSI that are rated on a four-point Likert scale (ranging from never to often). As described above, Nock and Prinstein [26] grouped the FASM items into four factors, which they termed: Automatic-Negative, Automatic-Positive, Social-Negative, and Social-Positive Reinforcement.

#### Alexian Brothers Urge to Self-Injure Scale (ABUSI)

The ABUSI assesses the frequency, intensity, and duration of the urge to self-injure, as well as the difficulty of resisting the urge and the overall urge or desire to engage in self-injury in the prior week. Responses are on a 7-point scale with a maximum total score of 30 and higher scores reflecting more intense urges to self-injure. The ABUSI demonstrates good psychometric properties in a sample of psychiatric patients treated for NSI [31]. For the present study the ABUSI will be used as an indicator of NSSI severity to evaluate the predictive validity of the functional factors. In this sample coefficient alpha for the ABUSI was very high (*α* = .93).

## Results

### ISAS and FASM structure

Exploratory factor analysis (EFA) was conducted in Mplus 7.31. Observed indicators were declared as categorical and we relied on the robust mean and variance adjusted weighted least squares estimator (WLSMV) for estimation. WLSMV includes all available data by relying on pairwise associations between variables to include cases with missing data. There were missing data for 199 cases for the ISAS (100 cases missing no more than 3 items), and for 26 cases on the FASM (18 missing no more than 2 items). EFA was chosen because of its utility for identifying the latent structure of a set of variables, as opposed to principal components analysis which is best suited for data reduction [30]. Oblique promax rotation was used to allow for the possibility that resulting factors would correlate. The number of factors to retain was based on an integration of considerations: inspection of the scree plot to identify the number of factors above the ‘elbow’, overlap or redundancy of factors, the conceptual interpretability of factors, and the size of eigenvalues/amount of variance explained for each factor [30]. Consistent with commonly followed recommendations [11], we opted to use .40 as a minimum factor loading to identify an item as belonging to a particular factor.

#### ISAS

**Fig. 1:**
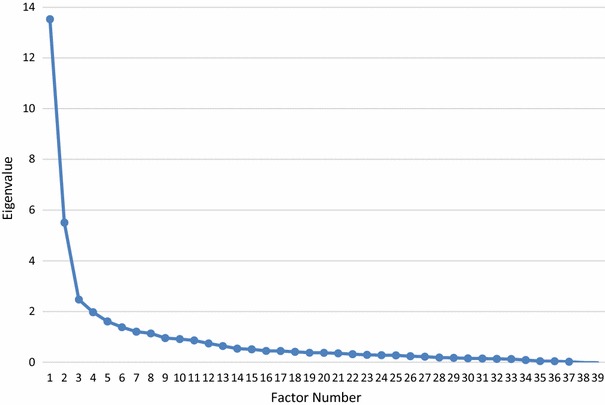
Scree plot for the exploratory factor analysis of the 39 ISAS items

For the 39 ISAS items, inspection of the scree plot and eigenvalues (see Fig. 1) indicated a two-factor solution accounting for 48.8 % of the total variance. Factor 1 had an eigenvalue of 13.5 and included Social functions, and Factor 2 had an eigenvalue of 5.5 and included Intrapersonal functions. The two factors yielded an intercorrelation of .39. As indicated in Table 1, 38 of 39 items-loadings were consistent with the scale loadings reported in Klonsky and Glenn [17]. One item (Item 17) loaded on the Intrapersonal rather than the Social factor. Summing the items belong to each factor resulted in scales with excellent internal consistencies as indexed by coefficient alpha: .88 for Intrapersonal and .89 for Social.

**Table 1:** Factor loadings of 39 Inventory of Statements About Self-injury (ISAS) items

ISAS item	ISAS Scale^a^	Original factor^a^	Intrapersonal (Factor 1)	Social (Factor 2)
1	Affect Regulation	Intrapersonal	.62	−.17
2	Interpersonal Boundaries	Social	.14	.51
3	Self-Punishment	Intrapersonal	.68	−.10
4	Self-Care	Social	.10	.55
5	Anti-Dissociation	Intrapersonal	.63	.03
6	Anti-Suicide	Intrapersonal	.78	−.07
7	Sensation-Seeking	Social	.11	.52
8	Peer-Bonding	Social	−.37	.90
9	Interpersonal Influence	Social	.12	.42
10	Toughness	Social	.25	.55
11	Marking Distress	Intrapersonal	.44	.34
12	Revenge	Social	−.19	.84
13	Autonomy	Social	.17	.63
14	Affect Regulation	Intrapersonal	.85	−.26
15	Interpersonal Boundaries	Social	.18	.65
16	Self-Punishment	Intrapersonal	.82	−.07
17	Self-Care	Social	.50	.26
18	Anti-Dissociation	Intrapersonal	.71	.12
19	Anti-Suicide	Intrapersonal	.79	−.04
20	Sensation-Seeking	Social	−.09	.80
21	Peer-Bonding	Social	−.33	.84
22	Interpersonal Influence	Social	.03	.59
23	Toughness	Social	.18	.66
24	Marking Distress	Intrapersonal	.46	.39
25	Revenge	Social	−.16	.89
26	Autonomy	Social	.25	.56
27	Affect Regulation	Intrapersonal	.87	−.26
28	Interpersonal Boundaries	Social	.22	.60
29	Self-Punishment	Intrapersonal	.84	−.10
30	Self-Care	Social	.17	.55
31	Anti-Dissociation	Intrapersonal	.59	.19
32	Anti-Suicide	Intrapersonal	.74	.00
33	Sensation-Seeking	Social	.08	.60
34	Peer-Bonding	Social	−.26	.91
35	Interpersonal Influence	Social	−.06	.68
36	Toughness	Social	.32	.64
37	Marking Distress	Intrapersonal	.56	.23
38	Revenge	Social	−.21	.72
39	Autonomy	Social	.17	.66

^a^Based on Klonsky and Glenn [17]

#### FASM

**Fig. 2:**
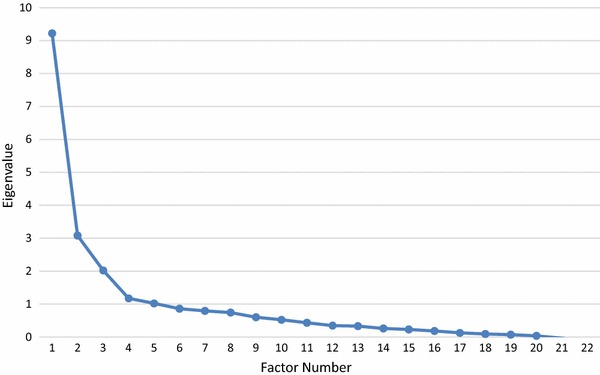
Scree plot for the exploratory factor analysis of the 22 FASM items

For the 22 FASM items, inspection of the scree plot and eigenvalues (see Fig. 2) indicated two possible solutions, a two-factor solution accounting for 55.9 % of the total variance and a three-factor solution accounting for 65.1 % of the total variance.

Regarding the two-factor solution, Factor 1 had an eigenvalue of 9.2 and included Social functions, and Factor 2 had an eigenvalue of 3.1 and included Intrapersonal functions. The two factors yielded an intercorrelation of .40. As indicated in Table 2, 19 of 22 items loaded on the superordinate Intrapersonal or Social factors in a manner consistent with the loadings reported in Nock and Prinstein [26]. Three items (Items 6, 9, and 18) loaded on the Intrapersonal rather than the Social factor. Summing the items belong to each factor resulted in scales with excellent internal consistencies as indexed by coefficient alpha: .79 for Intrapersonal and .89 for Social.

**Table 2:** Factor loadings of 22 Functional Assessment of Self-Mutilation (FASM) items

FASM item	FASM Scale^a^	Original factor^a^	Social (Factor 1)	Intrapersonal (Factor 2)
1	Social Negative	Social	.67	.12
2	Automatic Negative	Intrapersonal	−.04	.67
3	Social Positive	Social	.87	−.07
4	Automatic Positive	Intrapersonal	−.02	.76
5	Social Negative	Social	.65	.24
6	Social Positive	Social	.09	.58
7	Social Positive	Social	.89	−.04
8	Social Positive	Social	.97	−.17
9	Social Negative	Social	.38	.51
10	Automatic Negative	Intrapersonal	−.06	.62
11	Social Positive	Social	.61	.04
12	Social Positive	Social	.78	.12
13	Social Negative	Social	.54	.22
14	Automatic Negative	Intrapersonal	−.32	.80
15	Social Positive	Social	.59	.20
16	Social Positive	Social	.92	−.10
17	Social Positive	Social	.82	.01
18	Social Positive	Social	.26	.42
19	None	Social^b^	.80	−.26
20	Social Positive	Social	.63	.12
21	Social Positive	Social	.76	.01
22	Automatic Positive	Intrapersonal	−.09	.71

^a^Based on Nock and Prinstein [26]

^b^Although Nock and Prinstein [26] did not include this item in their factor analysis, we regarded the item-content (“to give yourself something to do with others”) as reflecting a social function

We also considered a three-factor solution because a third factor had an eigenvalue of 2.0 and appeared modestly above the elbow in the scree plot (Fig. 2). The three-factor solution turned out to be equivalent to the three-factor solution reported in Dahlström et al. [7]. One factor comprised the intrapersonal items (Items 2, 4, 6, 10, 14, 22), a second comprised items related to social influence (Items 3, 7, 8, 11, 15, 17, 20), and a third comprised items primarily related to peer identification (e.g., “to feel more a part of a group) but also avoidance (e.g., “to avoid punishment or paying the consequences”) and solitary behavior (e.g., “to give yourself something to do when alone”). The two social factors were highly correlated (*r* = .54). Because this third factor lacked clear conceptual coherence, was highly correlated with the social influence factor, and had the least empirical justification (small eigenvalue), we opted to retain the two-factor solution. However, the information we report regarding the third factor should be of use to readers who wish to consider the three-factor solution further, especially given its empirical convergence with Dahlström et al. [7].

### Predictive validity of the two-factor structure

Past research has found that endorsement of Intrapersonal functions relates to indicators of clinical severity more strongly than endorsement of Social functions [17, 27]. Therefore, we conducted post hoc analyses to examine the relationship of both the ISAS and FASM Intrapersonal and Social factors to two indicators of NSI severity: (1) frequency of NSI in the past week (as indicated in chart records), and (2) urge to self-injure (as measured by the ABUSI; [31]). Skewness and kurtosis were within normal limits for past week self-injury frequency, ABUSI, and both ISAS and FASM intrapersonal scales, but was high (>2.5) for the ISAS and FASM social scales. Therefore, these scales were rank-transformed, which reduced kurtosis to below an absolute value of 1.3 for both scales.

Consistent with previous research, Intrapersonal functions exhibited a general pattern of correlating more strongly with indicators of NSI severity (see Table 3). Specifically, both recent NSI frequency and urge correlated more strongly with ISAS Intrapersonal functions than with ISAS Social functions (*p*s ≤ .001). Similarly, NSI urge correlated more strongly with FASM Intrapersonal functions than FASM Social functions (*p* = .001). However, correlations of recent NSI frequency with FASM Intrapersonal and Social functions were similar in magnitude.

**Table 3:** Relations of Intrapersonal and Social functions to indicators of NSI severity

	ISAS Intrapersonal	ISAS Social	FASM Intrapersonal	FASM Social
NSI urge (ABUSI)	.50	.17	.48	.19
NSI frequency (past week)	.23	.15	.26	.25

All correlations significant at *p* < .05

## Discussion

This study examined the structure of NSI functions in adolescent and adult patients receiving acute-care treatment for NSI. Converging evidence from two different measures of NSI functions indicated that the functions of NSI are well captured by a two-factor structure. One factor represents Social functions, or social reinforcement of NSI (e.g., influencing others, facilitating peer-bonding), and a second factor represents Intrapersonal functions, or self-focused reinforcement of NSI (e.g., reducing one’s negative emotions, ending dissociative experiences). The two factors are moderately correlated (*r*s ≈ .4), indicating that they represent conceptually distinguishable constructs.

Findings suggest that the two-factor structure may best capture the structure of NSI functions across measurement tools. This study used two independently developed measures of NSI functions, and found that analyses of each measures were consistent with the two-factor structure of NSI. This pattern of converging evidence suggests that the two-factor structure is not merely an artifact of a specific measure’s design or content. Further, taken together with previous findings [17, 26], the two-factor structure has now been found in multiple settings (university, clinical) and samples (adolescents, young adults, adults), indicating that it is likely to generalize to diverse populations. Finally, in support of the construct validity of the two factor structure, this study replicated previous findings [17] that Intrapersonal functions of NSI are more strongly associated with clinical severity than Social functions.

While we emphasize evidence for the two-factor structure, it is important to note that the FASM might also be reasonably represented by a three-factor structure. The present study found empirical support for a three-factor structure equivalent to findings from a recent, large-scale study by Dahlström et al. [7] as well as a study by Young et al. [34]. Because this structure did not replicate in the ISAS, and because the third FASM factor included a variety of items that did not have obvious conceptual coherence yet maintained a high intercorrelation with the other factor containing social items, we felt the two-factor structure (Intrapersonal and Social) had the most conceptual and empirical support. However, it will be important for future studies utilizing confirmatory factor analysis to address this issue and directly compare fits between the two- and three-factor solutions.

Findings have implications for treatment and future research. Understanding the functions of NSI can be critical for treating individuals engaging in NSI. Identifying the relative importance and meanings of Intrapersonal versus Social functions of NSI can enrich case formulation and facilitate treatment decisions. For example, individuals with high endorsement of Intrapersonal functions may benefit from interventions that focus on affect regulation, and may require more intensive treatment and risk management. In contrast, individuals with high endorsement of Social functions may benefit from interventions that focus on developing effective interpersonal skills. Individuals high on both Intrapersonal and Social functions will likely require that treatment address both functions. Knowledge about functions can also inform future research seeking to develop new treatment approaches for NSI, and the possibility that treatment effectiveness may differ according to the functions present.

An important limitation of this study is the cross-sectional design. The correlations we found between Intrapersonal functions and clinical severity are consistent with previous research [17, 27], and suggest that endorsement of Intrapersonal functions may be indicative of NSI that is more persistent, less responsive to treatment, and more likely to progress to medically severe forms of self-injury, including suicide attempts. However, when it comes to understanding the prognostic and treatment implications of functions, longitudinal research will be required, and represents a clear next step. Indeed, cross-sectional correlates of NSI often fail to predict the behavior prospectively [10]. Future studies should therefore utilize the two-factor structure to examine the implications of NSI functions for key prognostic indicators (e.g., continuation of NSSI, maintenance and development of co-occurring psychopathology), as well as for the enhancement of treatment.

## Footnotes

1 For the remainder of the paper we use the term “Intrapersonal” to refer to what Nock and Prinstein [26] call Automatic functions and what Klonsky and Glenn [17] call Intrapersonal functions, and we use the term “Social” to refer to what Nock and Prinstein [26] call Social functions and what Klonsky and Glenn [17] call Interpersonal functions.
